# Continuous Renal Replacement Therapy With oXiris Filter May Not be an Effective Resolution to Alleviate Cytokine Release Syndrome in Non-AKI Patients With Severe and Critical COVID-19

**DOI:** 10.3389/fphar.2022.817793

**Published:** 2022-02-04

**Authors:** Kai Kang, Yunpeng Luo, Yang Gao, Jiannan Zhang, Changsong Wang, Dongsheng Fei, Wei Yang, Xianglin Meng, Ming Ye, Yan Gao, Haitao Liu, Xue Du, Yuanyuan Ji, Jieling Wei, Wanqiu Xie, Jun Wang, Mingyan Zhao, Kaijiang Yu

**Affiliations:** ^1^ Department of Critical Care Medicine, the First Affiliated Hospital of Harbin Medical University, Harbin Medical University, Harbin, China; ^2^ Department of Critical Care Medicine, the Sixth Affiliated Hospital of Harbin Medical University, Harbin Medical University, Harbin, China; ^3^ Institute of Critical Care Medicine, the Sino Russian Medical Research Center of Harbin Medical University, Harbin Medical University, Harbin, China; ^4^ Department of Critical Care Medicine, Harbin Medical University Cancer Hospital, Harbin Medical University, Harbin, China; ^5^ Department of Critical Care Medicine, the Second Affiliated Hospital of Harbin Medical University, Harbin Medical University, Harbin, China; ^6^ Department of Critical Care Medicine, the Fourth Affiliated Hospital of Harbin Medical University, Harbin Medical University, Harbin, China; ^7^ Key Laboratory of Hepatosplenic Surgery, Ministry of Education, Harbin, China; ^8^ The Cell Transplantation Key Laboratory of National Health Commission, Harbin, China

**Keywords:** continuous renal replacement therapy, oXiris filter, cytokine release syndrome, cytokine storm, hyper inflammation, non-acute kidney injury, severe and critical COVID-19, SARS-CoV-2 infection

## Abstract

In this study, we aimed to determine whether continuous renal replacement therapy (CRRT) with oXiris filter may alleviate cytokine release syndrome (CRS) in non-AKI patients with severe and critical coronavirus disease 2019 (COVID-19). A total of 17 non-AKI patients with severe and critical COVID-19 treated between February 14 and March 26, 2020 were included and randomly divided into intervention group and control group according to the random number table. Patients in the intervention group immediately received CRRT with oXiris filter plus conventional treatment, while those in the control group only received conventional treatment. Demographic data were collected and collated at admission. During ICU hospitalization, the concentrations of circulating cytokines and inflammatory chemokines, including IL-2, IL-4, IL-6, IL-10, TNF-α, and IFN-γ, were quantitatively measured daily to reflect the degree of CRS induced by SARS-CoV-2 infection. Clinical data, including the severity of COVID-19 white blood cell count (WBC), neutrophil proportion (NEUT%), lymphocyte count (LYMPH), lymphocyte percentage (LYM%), platelet (PLT), C-reaction protein (CRP), high sensitivity C-reactive protein (hs-CRP), alanine aminotransferase (ALT), aspartate aminotransferase (AST), total bilirubin (TB), albumin (ALB), serum creatinine (SCr), D-Dimer, fibrinogen (FIB), IL-2, IL-4, IL-6, IL-10, TNF-α, IFN-γ, number of hospital days and sequential organ failure assessment (SOFA) score were obtained and collated from medical records, and then compared between the two groups. Age, and SCr significantly differed between the two groups. Besides the IL-2 concentration that was significantly lower on day 2 than that on day 1 in the intervention group, and the IL-6 concentrations that were significantly higher on day 1, and day 2 in the intervention group compared to the control group, similar to the IL-10 concentration on day 5, there were no significant differences between the two groups. To sum up, CRRT with oXiris filter may not effectively alleviate CRS in non-AKI patients with severe and critical COVID-19. Thus, its application in these patients should be considered with caution to avoid increasing the unnecessary burden on society and individuals and making the already overwhelmed medical system even more strained (IRB number: IRB-AF/SC-04).

## Background

Excessive and uncontrolled systemic immune responses are the real culprit of disease deterioration and death in patients with Coronavirus disease 2019 (COVID-19), and not fatal virus infection (Gao et al., 2021). Severe acute respiratory syndrome coronavirus type 2 (SARS-CoV-2) infection can trigger the excessive production and release of a series of circulating cytokines and inflammatory chemokines, especially marked by IL-6, IL-10, and TNF-α, also known as cytokine release syndrome (CRS) or cytokine storm, which have been revealed to be closely associated with organ injury, disease severity and death in patients with COVID-19 (Copaescu et al., 2020; Del Valle et al., 2020; Huang et al., 2020). Cytokines concentrations in sputum and bronchoalveolar lavage fluid (BALF) are more representative than those in serum (Wang et al., 2021). However, short-term corticosteroid treatment based on the treatment concept of alleviating excessive and uncontrolled systemic immune responses is still full of controversy in clinical practice due to significant adverse effects in the middle and late stages of SARS-CoV-2 infection (van Paassen et al., 2020; Liu et al., 2021).

The therapeutic purpose of continuous renal replacement therapy (CRRT) has evolved from the single replacement of kidney function to support of multiple organ systems via the removal of circulating cytokines and inflammatory mediators. Accordingly, it can be considered for use in acute respiratory distress syndrome (ARDS) induced by current SARS-CoV-2 infection, especially for severe and critical COVID-19 patients with CRS, regardless of the presence of acute kidney injury (AKI) complications (Gao et al., 2019; Zhang et al., 2020; Xiang et al., 2021). oXiris filter is a highly biocompatible modified hemodiafilter with a special heparin-coated design, which can be combined with CRRT in clinical practice. CRRT with oXiris filter can further enhance the clearance of circulating cytokines and inflammatory chemokines, improve clinical symptoms and laboratory indicators, reduce disease severity, and prolong the survival time in critically ill patients with COVID-19 (Cascarano et al., 2021; Premužić et al., 2021; Rosalia et al., 2021). Still, the relevant studies mainly focused on acute or chronic renal failure in patients with COVID-19, which is one of the potential indications for CRRT initiation.

In our study, we tried to explore the role of CRRT with oXiris filter on CRS in non-AKI patients with severe and critical COVID-19 by comparing serum concentrations of cytokines and inflammatory chemokines during ICU hospitalization. Our findings will provide a solid theoretical basis that can guide its clinical application.

## Methods

### Study Design

A total of 17 non-AKI patients with severe and critical COVID-19 treated at COVID-19 treatment center of Heilongjiang province in the First Affiliated Hospital of Harbin Medical University between February 14 and March 26, 2020, were included in this prospective non-blind randomized controlled study. These patients were randomly divided into intervention group and control group according to the random number table. After randomization, patients in the intervention group immediately received CRRT with oXiris filter plus conventional treatment, while those in the control group only received conventional treatment. Based on the Diagnosis and Treatment of New Coronavirus Pneumonia (the fifth edition), conventional treatment included antiviral, aerosol inhalation, antibiotics for confirmed infected patients, different forms of oxygen therapy for patients with decreased blood oxygen saturation, analgesia and sedation for patients with relevant needs, etc., instead of immunomodulatory therapy such as corticosteroid and IL-6 monoclonal antibody. These patients were dealt with by the same group of experienced intensivists in the ICU.

Demographic data were collected and collated at admission. Clinical data, including the severity of COVID-19 white blood cell count (WBC), neutrophil proportion (NEUT%), lymphocyte count (LYMPH), lymphocyte percentage (LYM%), platelet (PLT), C-reaction protein (CRP), high sensitivity C-reactive protein (hs-CRP), alanine aminotransferase (ALT), aspartate aminotransferase (AST), total bilirubin (TB), albumin (ALB), serum creatinine (SCr), D-Dimer, fibrinogen (FIB), IL-2, IL-4, IL-6, IL-10, TNF-α, IFN-γ, and number of hospital days were obtained and collated from medical records during hospitalization, and then compared between the two groups. The serum concentrations of IL-2, IL-4, IL-6, IL-10, TNF-α, and IFN-γ were used as the primary research endpoint to reflect the degree of CRS induced by SARS-CoV-2 infection. Sequential organ failure assessment (SOFA) scores were calculated during the first 24 h clinical data after ICU admission.

The study was approved by the Ethics Committee of the First Affiliated Hospital of Harbin Medical University (IRB number: IRB-AF/SC-04).

### Study Population

In this study, the inclusion criteria were following: admitted to ICU; age ≥18 years old; confirmed severe and critical patients with COVID-19; non-AKI; written informed consent obtained from patients or guardians; whereas COVID-19 patients who met the following criteria were excluded: uncontrolled malignant tumors with multiple metastases; leukemia; acquired immunodeficiency syndrome (AIDS); obstructive pneumonia caused by pulmonary tumors, severe pulmonary interstitial fibrosis, pulmonary alveolar proteinosis, and allergic alveolitis; chronic organ failure; immunotherapy or organ transplant within 6 months; autoimmune disorder; need extracorporeal membrane oxygenation (ECMO) or extracorporeal carbon dioxide removal (ECCO2R) at ICU admission; patients expected to die within 72 h; pregnant or breastfeeding women; incomplete medical records; any potential conditions endangering the patient’s safety. The full panel of experts was responsible for identifying potential conditions that could endanger the safety of enrolled patients.

### Diagnosis of Severe and Critical COVID-19

All enrolled patients were confirmed by detection of SARS-CoV-2 nucleic acid on oropharyngeal swabs, nasopharyngeal swabs, or lower respiratory tract specimens, and then classified into severe or critical cases according to the Diagnosis and Treatment of New Coronavirus Pneumonia (the fifth edition).

### Measurement of Circulating Cytokines and Inflammatory Mediators

During ICU hospitalization, the serum concentrations of cytokines and inflammatory chemokines, including IL-2, IL-4, IL-6, IL-10, TNF-α, and IFN-γ, were quantitatively measured by cytometric bead array (CBA) on a daily basis.

### Continuous Renal Replacement Therapy With oXiris Filter

After randomization, CRRT with oXiris filter was immediately applied to patients in the intervention group. In this study, a temporary double-lumen central venous catheter (11.5 F) was used to establish vascular access under ultrasound guidance. Patients in the intervention group were managed with 72 h CVVH combined oXiris filter (Baxter International, Deerfield, IL, United States), regional citrate anti-coagulation, and a pre- and post-dilution ratio of 1:1 on a Prismaflex system (Baxter International, Deerfield, IL, United States). Blood flow rates, dehydration volume, and amount of substitute fluid were individually adjusted according to the different conditions and treatment needs of each patient. Considering the saturation of membrane adsorption, Oxiris filter was changed every 12 h.

### Data Collection

Demographic data, including age, gender, comorbidities, and clinical data, including the severity of COVID-19, SOFA score, WBC, NEUT%, LYMPH, LYM%, PLT, CRP, hs-CRP, ALT, AST, TB, ALB, SCr, D-Dimer, FIB, IL-2, IL-4, IL-6, IL-10, TNF-α, IFN-γ, and number of hospital days were obtained and collated from medical records during hospitalization by dedicated personnel in our research team. None of the other members of our research team was privy to enrolled patient’s personal information beyond what was required for this study.

### Statistical Analyses

SPSS 24.0 (SPSS, Inc., Chicago, IL) was used for statistical analyses. Continuous data conforming to normal distribution were described as mean ± standard deviation (SD), while continuous data with non-normal distribution were expressed as median (P25, P75). The measurement data were expressed by frequency. Independent-samples *t*-test was used to perform inter-group comparison for continuous data with normal distribution, while Mann-Whitney *U* test was employed for inter-group comparison of abnormally distributed continuous data. The Fisher’s exact test was used for comparing measurement data between the two groups. *p*-values <0.05 were considered to indicate statistical significance.

## Results

### Comparison of Demographic and Clinical Baseline Data Between the Two Groups

**FIGURE 1:**
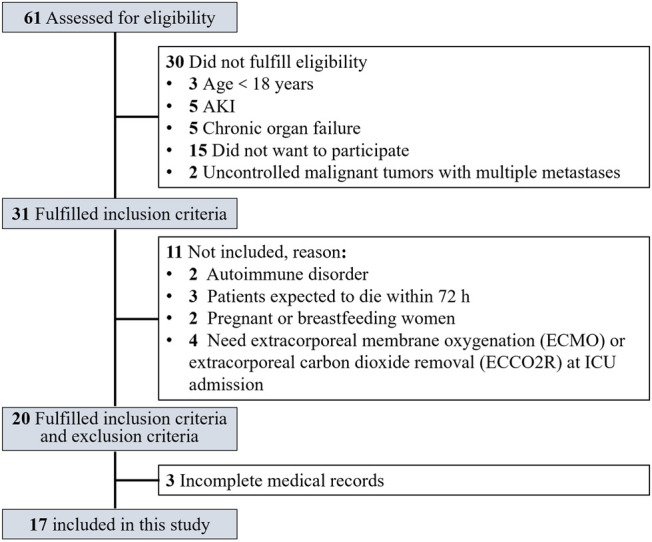
Flowchart of study participants.

Flowchart of study participants was shown in Figure 1. A total of 17 non-AKI patients with severe and critical COVID-19 treated between February 14 and March 26, 2020 were included and randomly divided into intervention group and control group according to the random number table. As shown in Table 1, age and SCr were significantly different in the two groups (*p* = 0.026, = 0.049, respectively), despite the randomization process, while no significant difference was observed in the remaining demographic and clinical baseline data.

**TABLE 1:** Comparison of demographic and clinical baseline data between the two groups.

	Intervention group	Control group	*t/z/x* ^*2*^	*P*
Age	67.63 ± 9.87	56.22 ± 9.16	2.47	0.026
Gender (male/female)	7/1	6/3	—	0.576
Hypertension (yes/no)	2/6	4/5	—	0.620
Diabetes (yes/no)	0/8	2/7	—	0.471
Severity of COVID-19 (severe/critical)	7/1	8/1	—	1.000
SOFA score	4 (3, 4)	4 (2.5, 4.5)	−0.31	0.754
WBC	6.48 ± 2.22	7.69 ± 2.32	−1.10	0.289
NEUT%	80.98 ± 9.71	85.38 ± 5.18	−1.15	0.278
LYMPH	10.68 ± 7.46	8.01 ± 3.84	0.91	0.384
LYM%	0.62 ± 0.34	0.55 ± 0.16	0.47	0.647
PLT	244.50 ± 150.99	247.89 ± 61.98	−0.06	0.951
CRP	46.58 ± 25.65	58.78 ± 58.26	−0.55	0.593
hs-CRP (> 10 mg/L/other)	7/1	8/1	—	1.000
ALT	26.41 (23.83, 77.05)	36.35 (18.65, 58.90)	−0.10	0.923
AST	40.59 ± 24.98	37.39 ± 27.02	0.25	0.804
TB	18.37 ± 12.27	30.13 ± 18.83	−1.50	0.154
ALB	27.67 ± 5.39	28.28 ± 2.27	−0.30	0.770
SCr	71.00 ± 17.97	54.73 ± 13.30	2.138	0.049
D-Dimer	1.75 (1.38, 2.63)	1.93 (1.18, 25.40)	−0.48	0.630
FIB	5.84 ± 1.30	3.96 ± 2.22	2.10	0.053
IL-2 level at admission	1.10 (0.91, 1.15)	1.19 (1.10, 1.23)	−1.26	0.207
IL-4 level at admission	0.59 ± 0.32	1.01 ± 0.76	−1.54	0.153
IL-6 level at admission	13.88 (2.67, 48.04)	10.46 (3.68, 25.26)	−0.58	0.564
IL-10 level at admission	6.71 ± 3.97	7.00 ± 3.40	−0.16	0.873
TNF-α level at admission	0.16 (0.01, 0.49)	0.05 (0, 0.56)	−0.30	0.767
IFN-γ level at admission	0.99 ± 0.11	1.19 ± 0.81	−0.73	0.483
Hospital day	28.63 ± 6.86	28.22 ± 6.38	0.13	0.902

### Comparison of IL-2, IL-4, IL-6, IL-10, TNF-α, and IFN-γ Concentrations Between the Two Groups From Day 1 to Day 6

**FIGURE 2:**
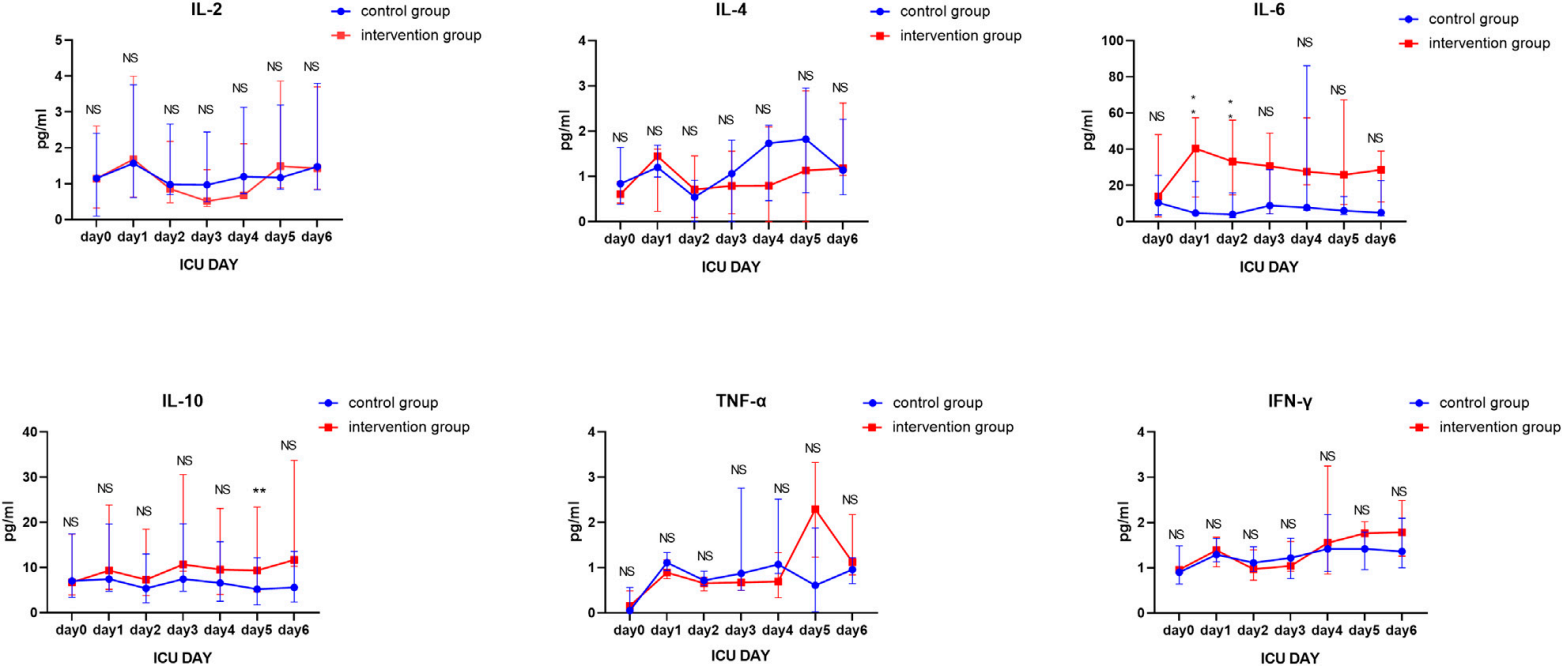
Longitudinal comparison of IL-2, IL-4, IL-6, IL-10, TNF-α, and IFN-γ concentrations between the two groups from day 1 to day 6.

As shown in Table 2 and Figure 2, besides the IL-2 concentration that was significantly lower on day 2 than that on day 1 in the intervention group, and the IL-6 concentrations that were significantly higher on day 1, and day 2 in the intervention group compared to the control group, similar to the IL-10 concentration on day 5, there were no significant differences between the two groups.

**TABLE 2:** Comparison of IL-2, IL-4, IL-6, IL-10, TNF-α, and IFN-γ concentrations between the two groups from day 1 to day 6.

	Group	Day 1	Day 2	Day 3	Day 4	Day 5	Day 6	X^2^_Mauchy_/P	F_(G-G)_/P	F_group_/P	F_group*time_/P
IL-2	Control group	1.5 ± 0.62	0.98 ± 0.70	0.97 ± 0.50	1.20 ± 0.73	1.17 ± 0.85	1.48 ± 0.84	27.95/0.016	3.98/0.012	1.07/0.318	0.85/0.745
	Intervention group	1.68 ± 0.63	0.86^a^ ± 0.47	0.52 ± 0.37	0.68 ± 0.76	1.49 ± 0.89	1.43 ± 0.84				
IL-4	Control group	1.36 ± 0.44	0.55 ± 0.49	0.94 ± 1.05	1.41 ± 0.89	1.77 ± 1.19	1.35 ± 0.93	32.78/0.004	1.85/0.152	0.03/0.861	0.39/0.762
	Intervention group	1.08 ± 0.70	0.78 ± 0.68	1.07 ± 1.13	1.17 ± 1.42	1.38 ± 1.41	1.70 ± 1.21				
IL-6	Control group	1.99 ± 0.99	1.22 ± 2.37	2.31 ± 0.99	2.82 ± 1.46	2.04 ± 0.91	2.07 ± 1.22	20.54/0.119	1.91/0.103	5.97/0.027	1.71/0.177
	Intervention group	3.41^a^ ± 0.78	3.25^a^ ± 0.99	2.93 ± 1.34	3.42 ± 0.62	3.24 ± 1.15	3.20 ± 1.08				
IL-10	Control group	7.45 ± 4.75	5.39 ± 2.19	7.46 ± 4.75	6.59 ± 2.53	5.20 ± 1.74	5.57 ± 2.40	30.71/0.007	0.37/0.521	6.18/0.025	0.47/0.676
	Intervention group	9.33 ± 5.18	7.32 ± 3.80	10.67 ± 9.19	9.54 ± 4.01	9.37^a^ ± 4.65	11.70 ± 10.29				
TNF-α	Control group	1.14 ± 0.26	0.73 ± 0.36	1.50 ± 1.53	1.50 ± 1.10	0.90 ± 1.10	1.02 ± 0.62	42.03/0.000	2.10/0.116	0.003/0.959	1.86/0.316
	Intervention group	0.93 ± 0.24	0.65 ± 0.18	0.69 ± 0.41	0.82 ± 0.55	2.36 ± 1.59	1.40 ± 0.73				
IFN-γ	Control group	1.39 ± 0.32	1.14 ± 0.34	1.26 ± 0.57	1.52 ± 0.68	1.44 ± 0.61	1.44 ± 0.88	110.44/0.000	2.04/0.162	1.61/0.223	1.23/0.300
	Intervention group	1.34 ± 0.37	1.07 ± 0.38	1.22 ± 0.47	2.04 ± 1.51	2.27 ± 1.95	3.10 ± 4.00				

a Represent significant difference compared with the control group.

## Discussion

As a novel, highly pathogenic human coronavirus (hCoV), SARS-CoV-2 may continue to pose a persistent and unprecedented threat to global public health security for a considerable time to come. Breakthrough infections caused by adaptive mutations in the SARS-COV-2 genome prevent universal vaccination from becoming the effective coping strategy against COVID-19 (Hacisuleyman et al., 2021). In the absence of available targeted interventions, there is an urgent need to explore effective treatment approaches based on an ongoing understanding of the pathogenesis of SARS-CoV-2 viral infection and disease deterioration. Among them, CRRT with oXiris filter is a highly expected and promising clinical approach (Chen et al., 2020).

Data from the Chinese Center for Disease Control and Prevention indicated that the mortality of patients with critical COVID-19 was close to 50% and even exceeded 60% in the early stages of the outbreak (Yang et al., 2020). Some of the survivors, who recovered from severe or critical COVID-19, were reported to suffer from severely impaired pulmonary diffusion capacities and abnormal chest imaging manifestations at 6-months follow-up after SARS-CoV-2 infection (Huang et al., 2021). Although the exact pathogenesis of SARS-CoV-2 infection and disease deterioration is still poorly understood, CRS characterized by an excessive and uncontrolled systemic inflammatory response has an essential role (Mehta et al., 2020). A significant increase in circulating cytokines and inflammatory chemokines burden was observed in patients with COVID-19, in association with multiple-organ dysfunction, increased disease severity, and adverse clinical outcomes (Huang et al., 2020; Zeng et al., 2020; Sims et al., 2021). Regulating inflammatory responses to restore immunological equilibrium and maintain immune homeostasis may be an entry point.

Using CRRT for immunomodulation has a long history in clinic for tapering cytokine storms and controlling the associated dysregulation of the immune system. This approach has also been proposed as adjuvant therapy in many diseases, including sepsis (Monard et al., 2019), septic AKI (Turani et al., 2019), septic shock (Schwindenhammer et al., 2019), severe Middle East Respiratory Syndrome (MERS) (Cha et al., 2015), severe acute pancreatitis (SAP) with or without ARDS (Cui et al., 2014; Gao et al., 2018), CRS induced by some immunotherapies (Constantinescu et al., 2020), severe burns (Peng et al., 2005), etc. Yet, a non-selective clearance allows harmful and beneficial substances to be simultaneously removed during CRRT (Shi et al., 2021). Moreover, the potential disadvantages of CRRT, such as technical complications of establishing vascular access, anti-coagulation-related complications, hemodynamic instability, internal environment disturbance, obstacles to the spontaneous recovery of renal function, and huge cost, should not be ignored in clinical practice. As a result, although some studies have confirmed the immunomodulation effect of CRRT, this was not sufficient to influence clinical endpoints and could even prolong the need for organ support (Park et al., 2016; Yin et al., 2020).

oXiris filter is superficially modified from an AN-69 membrane (polyacrylonitrile) with an additional positively charged polyimide ethylene layer used to enhance the cytokines-adsorbing capacity by ionic bonding and grafted with heparin (Broman et al., 2019), which was first approved and marketed in Europe in 2009. The addition of the highly adsorptive preheparinized oXiris filter can enhance the ability of CRRT to effectively remove endotoxin, circulating cytokines, and inflammatory chemokines, thus reducing lactate concentration and vasopressors infusion rate, and improving haemodynamic status, systemic perfusion, multi-organ function, and clinical outcomes without related adverse events (Broman et al., 2019; Schwindenhammer et al., 2019; Turani et al., 2019; Villa et al., 2020; Samman et al., 2021). Therefore, it gained emergency approval from US Food and Drug Administration (FDA) for the clinical treatment of COVID-19 in April 2020 to counter the CRS attack triggered by SARS-CoV-2 infection. Different from short-term corticosteroid treatment, CRRT with oXiris filter is expected to mitigate circulating cytokines and inflammatory chemokines burden and restore immune homeostasis in COVID-19 patients without a prolonged state of immunosuppression and serious secondary infection.

In our study, CRRT with oXiris filter showed no advantage in removing circulating cytokines and inflammatory chemokines in non-AKI patients with severe and critical COVID-19. The primary reason is that all the selected patients were non-AKI with normal renal clearance, which is significantly different from previous relevant studies that mainly focused on the acute or chronic renal failure patients with seriously damaged renal clearance. In such case, CRRT with or without oXiris filter becomes an important way to clear circulating cytokines and inflammatory chemokines. In addition, the concentrations of IL-6 in patients with severe COVID-19 was usually tens of pg/mL, which was similar to our results and far lower than those in patients with septic shock or CRS induced by Chimeric Antigen Receptor (CAR) T-cell infusion (Aziz et al., 2020; Monneret et al., 2021). A previous study has confirmed that the immunomodulatory treatment of Afelimomab exerts a protective effect in patients with severe sepsis only when the concentration of IL-6 exceeds the threshold of 1,000 pg/ml (Panacek et al., 2004). The concentration of IL-6 in our study, which was far from reaching or close to this threshold, may explain these negative results. Furthermore, different inflammatory subphenotypes of COVID-19 may have a certain impact on the production and release of circulating cytokines and inflammatory chemokines (Lin et al., 2020). Lastly, changes in circulating cytokines and inflammatory chemokines concentrations may not be solely influenced by extracorporeal removal, but also by SARS-CoV-2 viral load, endogenous production, innate and acquired immune defense, comorbidities and many other factors (Bermejo-Martin et al., 2020; Rajpal et al., 2020; Lumlertgul et al., 2021; Pasrija and Naime, 2021).

There are some limitations in the present study. First of all, as this was a small-size single-center study, the possible positive results may be emerge after increasing the number of cases, and the credibility and generalizability of our conclusion may weaken. Second, the duration varies from onset to ICU admission, which may be influence the production and removal of circulating cytokines and inflammatory chemokines to a certain extent, although no significant difference was observed in the clinical baseline data between the two groups except Scr. Third, the monitoring duration of circulating cytokines and inflammatory chemokines concentrations was limited to 6 days, and thus the medium and long term role of CRRT with oXiris filter in non-AKI patients with severe and critical COVID-19 was not further explored. Finally, the types of circulating cytokines and inflammatory chemokines detection were also limited.

## Conclusion

CRRT with oXiris filter may not be an effective method for alleviating CRS in non-AKI patients with severe and critical COVID-19; thus, its application in these patients should be considered with caution to avoid increasing the unnecessary burden on society and individuals and making the already overwhelmed medical system even more overstretched. The findings of our study need to be further confirmed by well-designed large-sample studies.

## Data Availability

The raw data supporting the conclusion of this article will be made available by the authors, without undue reservation.
